# PZR promotes tumorigenicity of lung cancer cells by regulating cell migration and invasion via modulating oxidative stress and cell adhesion

**DOI:** 10.18632/aging.204771

**Published:** 2023-06-06

**Authors:** Ying Fu, Yuan Sui, Yuming Zhao, Jianzhuo Jiang, Xueyuan Wang, Jiarui Cui, Xueqi Fu, Shu Xing, Zhizhuang Joe Zhao

**Affiliations:** 1 Edmond H. Fischer Signal Transduction Laboratory, School of Life Sciences, Jilin University, Changchun, China; 2 Department of Laboratory Medicine, Jilin Medical University, Jilin, China; 3 Department of Pathology, University of Oklahoma Health Sciences Center, Oklahoma City, OK 73104, USA

**Keywords:** PZR, lung cancer, migration, tumorigenicity, oxidative stress

## Abstract

PZR is a transmembrane glycoprotein encoded by the *MPZL1* gene. It serves as a specific binding protein and substrate of tyrosine phosphatase SHP-2 whose mutations cause developmental diseases and cancers. Bioinformatic analyses of cancer gene databases revealed that PZR is overexpressed in lung cancer and correlated with unfavorable prognosis. To investigate the role of PZR in lung cancer, we employed the CRISPR technique to knockout its expression and recombinant lentiviruses to overexpress it in lung adenocarcinoma SPC-A1 cells. While knockout of PZR reduced colony formation, migration, and invasion, overexpression of PZR had the opposite effects. Furthermore, when implanted in immunodeficient mice, PZR-knockout SPC-A1 cells showed suppressed tumor-forming ability. Finally, the underlying molecular mechanism for these functions of PZR is its positive role in activating tyrosine kinases FAK and c-Src and in maintaining the intracellular level of reactive oxygen species (ROS). In conclusion, our data indicated that PZR plays an important role in lung cancer development, and it may serve as a therapeutic target for anti-cancer development and as a biomarker for cancer prognosis.

## INTRODUCTION

Protein Zero Related (PZR), also known as *MPZL1* for myelin protein zero-like protein 1, is an immunoglobulin superfamily transmembrane protein [1–3]. Its extracellular portion has about 40% of sequence homology to myelin P_0_, while its intracellular segment has two immunoreceptor tyrosine-based inhibition motifs (ITIMs) that specifically interact with SHP-2, a tyrosine phosphatase involved in a multitude of cellular processes including cancer development, metabolism, and responses to oxidative stress [4–8]. Tyrosyl phosphorylation of PZR has been reported to be associated with Noonan syndrome (NS) and LEOPARD syndrome (LS) [9, 10]. As a cellular adhesion molecule, PZR has been shown to be associated with leukocyte trafficking and to play an essential role in tumor metastasis to non-CNS sites [11]. Overexpression and tumor-promoting activities of PZR have been observed in many types of cancers including advanced gallbladder carcinoma, ovarian cancer, and hepatocellular carcinoma [12–14]. However, the potential function of PZR in lung cancer is not well defined.

Lung cancer is a deadly disease accounting for nearly a quarter of all cancer-related deaths [15]. The two major forms of lung cancer are non–small-cell lung cancer (NSCLC about 85% of all lung cancers) and small-cell lung cancer (SCLC about 15%) [15, 16]. NSCLC can be divided into three major histologic subtypes: squamous-cell carcinoma, adenocarcinoma, and large-cell lung cancer. Within NSCLC classifications, adenocarcinomas are the most common subtype of lung cancer. High levels of reactive oxygen species (ROS) are beneficial to tumor cells to maintain their proliferation, while effective removal of excessive ROS in tumor cells, changing the intracellular REDOX state, and inducing cells to carry out signal transduction pathways involving low levels are likely to inhibit tumor cell growth [7]. To investigate the biological function of PZR in lung adenocarcinoma, we analyzed existing cancer gene databases and found that PZR is overexpressed in lung cancer and serves as an unfavorable prognostic biomarker. We altered PZR expression in lung adenocarcinoma SPC-A1 cells and further characterized the effects *in vitro* and *in vivo*. Our data suggest that loss of PZR decreased migration, invasion, and the level of ROS in SPC-A1 cells *in vitro* and suppressed tumor growth *in vivo*.

## MATERIALS AND METHODS

### Databases

Gene Expression Profiling Interactive Analysis (GEPIA) (http://gepia.cancer-pku.cn/) [17], a web-based tool to analyze cancer and normal gene expressions in the TCGA and GTEx databases, was employed in the present study.

### Cell culture

Human lung cancer SPC-A1 cells were purchased from the Type Culture Collection of the Chinese Academy of Sciences (Shanghai, China) and cultured in RPMI-1640 medium supplemented with 10% fetal bovine serum and 1% penicillin/streptomycin at 37°C in a 5% CO_2_ atmosphere. Stable PZR-knockout and PZR-overexpressing lines derived from the parental SPC-A1 cells were cultured under the same condition. These cells were authenticated by GENEWIZ, Inc. (Suzhou, China) with 100% STR matching profiles. The cells were also regularly examined to ensure the absence of mycoplasma contaminations.

### Generation of PZR knockout SPC-A1 cells

Two sets of guide DNA primers targeting the second exon of PZR were designed using a web-based tool (http://crispor.tefor.net/). With sequences of CACCGACAGTAGTGTCGGCCCCCTC and CACCGTTCAAGTCTACTAGTACGAC, the primers were synthesized and cloned into the pSpCas9 (BB)-2A-Puro (PX459) V2.0 vector. The resulting plasmids were verified by DNA sequencing and then used to co-transfect SPC-A1 cells with lipofectamine 2000 (Invitrogen). Transfected cells were treated with 2 μg/mL puromycin and further subjected to clonal selection. Positive clones were verified by Western blotting analysis with an anti-PZR antibody, PCR amplification with primers GCTGGAGTATCAGCCTTGGAA (Pf. forward) and AGAAACCTGTGAGGCAGGACT (Pr. reverse), and subsequent DNA sequencing.

### Overexpression of PZR in SPC-A1 cells

The full-length PZR (Gene ID: 9019, NM_003953. 6) cDNA was sub-cloned to an LV 18 vector (GenePharma, Shanghai, China). The construct was then transfected to 293T cells together with the lentiviral packaging plasmids to generate PZR-expressing lentivirus (PZR-OE). Following filtration and enrichment, recombinant lentiviruses were used to infect SPC-A1 cells in the presence of 5 μg/mL polybrene. Thereafter, puromycin (2 μg/mL) was added to select stable cells. PZR overexpression was verified by Western blotting assays. Control cells designated V-OE were generated by infecting SPC-A1 cells with lentiviruses carrying the empty LV 18 vector.

### Western blotting

Cells were washed twice with ice-cold PBS after reaching near confluency and harvested by use of the RIPA lysis buffer and protease/phosphatase inhibitor (Servicebio) and Cocktail, 50× (Servicebio), and their concentrations were detected by BCA (Thermo Fisher Scientific). Cell extracts containing equal amounts of total proteins were separated by 4%–20% SDS-PAGE (Super-PAGE™ Bis-Tris Gels) and transferred to a polyvinylidene fluoride (PVDF) membrane for Western blotting with different primary antibodies and then with secondary antibodies. Detection was performed by using the enhanced chemiluminescence (ECL) method with the Tanon-5200 imaging system (Beijing YuanPingHao Biotech Co., Ltd.). Antibodies against PZR (#9893), cortactin (#3503), phospho-cortactin (Y421) (#4569), FAK (#71433), phospho-FAK (Y397) (#8556), c-Src (#2109), and phospho-c-Src (Y416) (#59548), β-actin (#4967) were all from Cell Signaling Technology. Secondary antibodies coupled to horseradish peroxidase were obtained from Absin (abs20040ss).

### Cell colony formation assays

Cells were seeded in 6-well plates at a density of 500 cells per well and cultured at 37°C for 7–10 days. Thereafter, the cells were fixed with 4% paraformaldehyde and stained with 0.1% (W/V) crystal violet. Pictures were taken, and megascopic cell colonies were counted using the Image J software. Each measurement was performed in triplicates, and the experiments were conducted at least three times.

### Wound healing assays

Cells were cultured in RPMI-1640 in a 6-well plate until reaching approximately 90% confluency and then scraped vertically with a 10 μL pipette tip to produce a cell-free area on the bottom of the plate. The cells were washed twice with PBS to remove detached cells and further cultured in RPMI-1640 without serum. Images along the scraped lines were captured at 0 h, 6 h, 24 h, and 48 h. Cell migration was calculated by using the Image J software.

### Cell invasion assays

Transwell upper chambers (6.5 mm membrane diameter, 8-μm pore size, Corning, USA) were coated with Matrigel matrix (30-fold dilution) and seeded with 1-2 × 10^4^ SPC-A1 cells. The lower chambers were filled with 600 μL complete culture medium containing 10% FBS. After 24 h of incubation, non-invading cells inside of the upper chambers were removed, and remaining cells on the membrane were fixed with 4% paraformaldehyde for 30 min, followed by staining with 0.1% (W/V) crystal violet for an additional 10 min. Pictures were taken under an inverted microscope, and cells were counted.

### Subcutaneous xenograft tumor mouse model

We used immunodeficient NYG mice to investigate the tumorigenicity of PZR-KO SPC-A1 cells. These mice were generated by knocking out Prkdc and Il2rg in Nucleotide binding oligomerization domain containing (NOD) mice using the CRISPR-Cas9 genome editing technique at Nanjing Medical University [18]. The mice were purchased from Liaoning Changsheng Biotechnology Co, Ltd, and kept under specific pathogen-free conditions (23 ± 2°C, 12 h light-dark cycle) with sterilized food and tap water given *ad libitum*. After washing with RPMI, 1 × 10^6^ trypsinized wild-type and PZR-knockout SPC-A1 cells in 0.1 mL incomplete medium were implanted in the flank of mice (male, 7 weeks old). The tumors were measured with a caliper every 4 days starting from the 5th day, and the tumor volume is calculated by using the formula V = 0.5 × ab^2^ (a = largest diameter, b = smallest diameter). Mice were euthanized by CO_2_ inhalation at the end of the experiment. Tissues and tumors were collected and subjected to fixation with formalin for histochemical staining. The size and weight of tumors were recorded. The patients/participants provided their written informed consent to participate in this study. The animal experiments were carried out under protocol No. YN2021097 approved by the Institutional Animal Care and Use Committee, School of Life Sciences, Jilin University, China.

### Histochemistry and immunostaining

Formalin-fixed, paraffin-embedded (FFPE) human and mouse tissue sections (4 μm) were subjected with hematoxylin/eosin (H/E) staining and immunohistochemical staining with antibodies against PZR and Ki67 following standard protocols. For immunofluorescence staining of lung cancer tissues, TRITC-labeled goat anti-rabbit 2nd antibody used was after probing of heat-treated sections with anti-PZR primary antibody.

### Detection of intracellular ROS levels

The degree of overall oxidative stress was determined by using a Reactive Oxygen Species Assay kit from Wanleibio (WLA131, Shenyang, China). In brief, SPC-A1 cells were incubated with 10 μM 2′, 7′-d dichlorodihydrofluorescein diacetate (DCFH-DA), a ROS indicator, for 30 min at 37°C in the dark. The green fluorescence was then observed under a fluorescence microscope or analyzed using a flow cytometer.

### Assay of antioxidant enzyme activity

The Superoxide Dismutase Detection Kit (A001; Nanjing Jiancheng Bioengineering Institute, Nanjing, China) was selected for SOD measurement. The assay was conducted according to the manufacturer’s instructions. Total superoxide dismutase (T-SOD) activity was assayed using the xanthine/xanthine oxidase method based on the production of O^2−^ anions.

### Statistical analysis

All measurement data were expressed as mean ± standard deviation (mean ± SD) and compared by *t*-test using the GraphPad Prism 9.0 software. *P* values of less than 0.05 are considered significantly different.

### Data availability

All data generated or analyzed during this study are available in this article.

## RESULTS

### PZR expression predicts unfavorable prognosis and is enhanced in lung cancer

**Figure 1:**
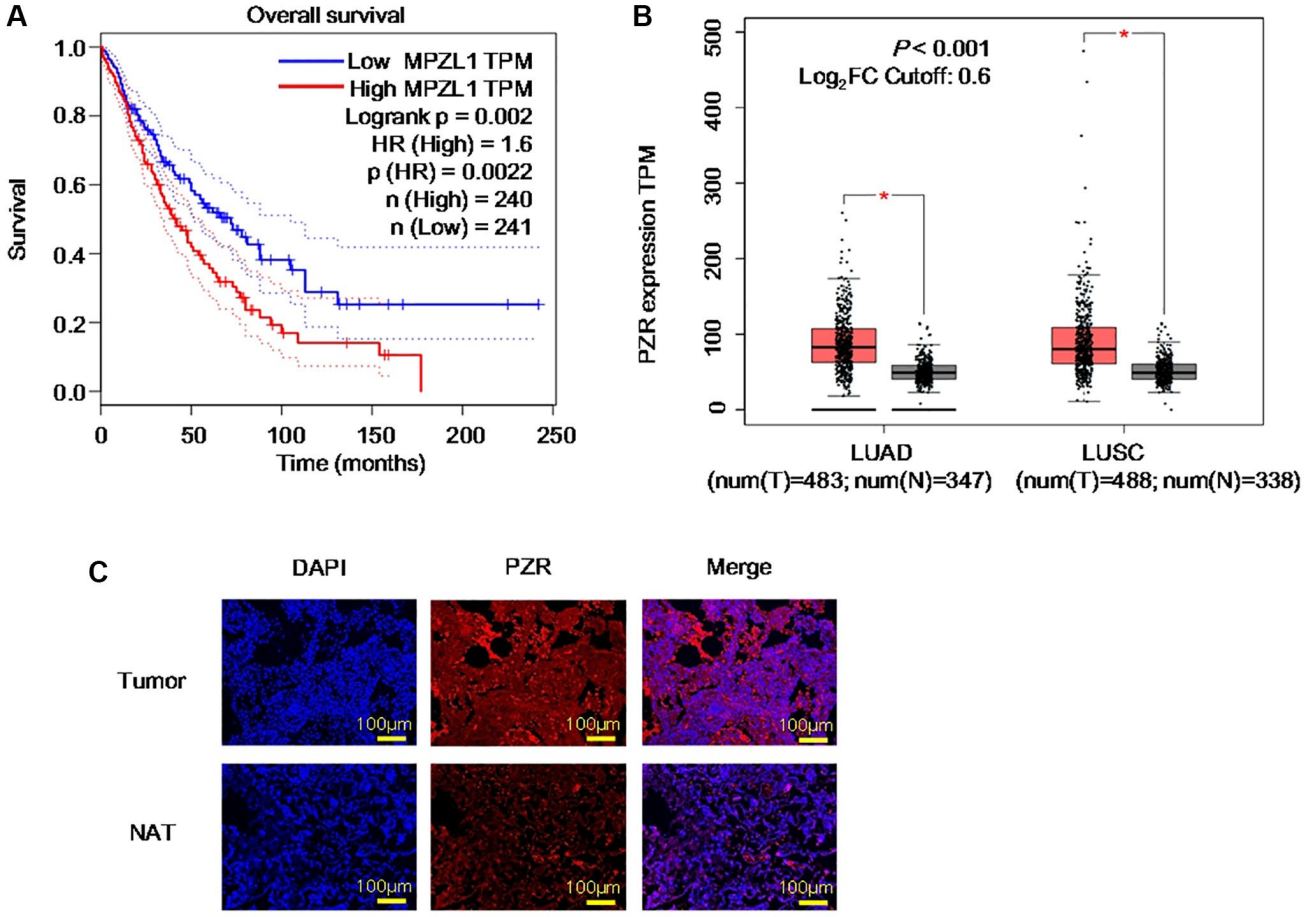
**PZR represents an unfavorable prognostic biomarker and is overexpressed in lung cancer.** (**A**) Expression data were from the TCGA databases and were analyzed by using the GEPIA tools. Overall survival analysis of TCGA lung adenocarcinoma (LUAD) and squamous cell carcinoma (LUSC) data via the GEPIA portal. (**B**) Comparison of PZR expression in normal and cancer lung tissues. Gene expression analysis of TCGA and GTEx databases LUAD and LUSC data via the GEPIA portal. (**C**) Immunofluorescent analyses PZR expression in lung cancer tissues and normal tissues adjacent to the tumor (NAT) (magnification, ×100). Data represent samples from 3 patients.

PZR was reported to be highly expressed in human advanced gallbladder carcinoma, ovarian cancer, and hepatocellular carcinoma and to play an important role in tumor development [14, 19, 20]. As a widely expressed protein, we thought that it may be involved in NSCLC, the major form of lung cancer and the deadliest worldwide [21]. By analyzing the TCGA database via the Gene Expression Profiling Interactive Analysis (GEPIA) portal, we found that high PZR expression is correlated with unfavorable prognosis with a hazards ratio of 1.6 and log rank *P* value of 0.002 (Figure 1A). Further analyses of the TCGA and GTEx gene expression databases revealed that PZR is overexpressed in both lung adenocarcinoma (LUAD) and lung squamous cell carcinoma (LUSC) in comparison with normal control lung tissues (Figure 1B). To verify the expression of PZR, we performed immunofluorescence staining of 3 pairs of matched malignant lung adenocarcinoma cancer tissues and adjacent normal tissues. The data demonstrated clearly higher levels of PZR in cancer cells (Figure 1C). Taken together, these findings suggest that PZR may play an important role in lung cancer development.

### Generation of PZR-knockout SPC-A1 cells

**Figure 2:**
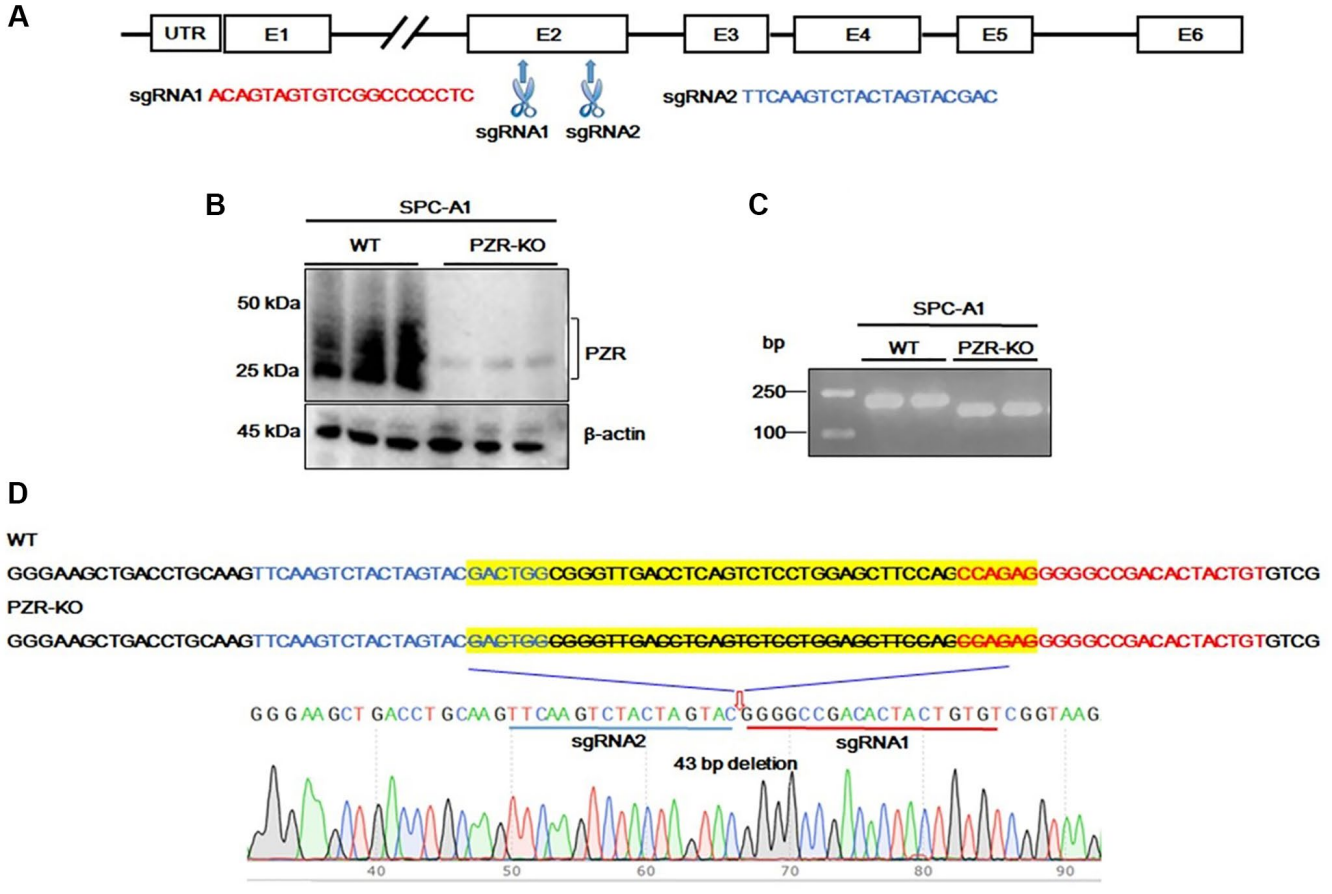
**Generation of PZR-knockout SPC-A1 cells.** (**A**) Two sgRNAs targeting exon 2 of the *MPZL1* gene were designed for CRISPR genome editing. (**B**) Verification of PZR knockout in SPC-A1 cells by Western blotting using antibodies against PZR and β-actin as indicated. (**C**) PCR analysis of genomic DNA from wild type and PZR-KO SPC-A1 cells primers Pf. and Pr. surrounding the sgRNA-targeting sites. PCR products were analyzed on 2% agarose and visualized with ethidium bromide. (**D**) DNA sequencing verification of a 43-bp deletion in PZR-KO SPC-A1 cells. Sequencing was performed from the 3′-side with primer Pr. The position of a 43-bp fragment deletion was marked by a red arrow.

The finding that high levels of PZR expression were associated with shorter overall survival in lung cancer prompted us to conduct cell-based mechanistic studies. For this purpose, we employed the human lung adenocarcinoma SPC-A1 cell line. First, we designed two sets of sgRNAs (PZR-sgRNA1 and PZR-sgRNA2) targeting different regions in exon 2 of *PZR* as shown in Figure 2A. Following cell transfection, we obtained multiple clonal cell lines lacking PZR expression as demonstrated by Western blotting analyses (Figure 2B). PCR amplification of PZR-knockout cell lines with primers surrounding exon 2 yielded a shorter product as resolved on agarose gels (Figure 2C). Further DNA sequencing analysis of the PCR product revealed a 43-bp deletion occurred at the predicated Cas9 cleavage sites (Figure 2D). This 43-bp deletion in exon 2 represents a frame shift mutation and lead to loss of PZR expression.

### Knockout of PZR reduces proliferation, migration, and invasion of SPC-A1 cells

**Figure 3:**
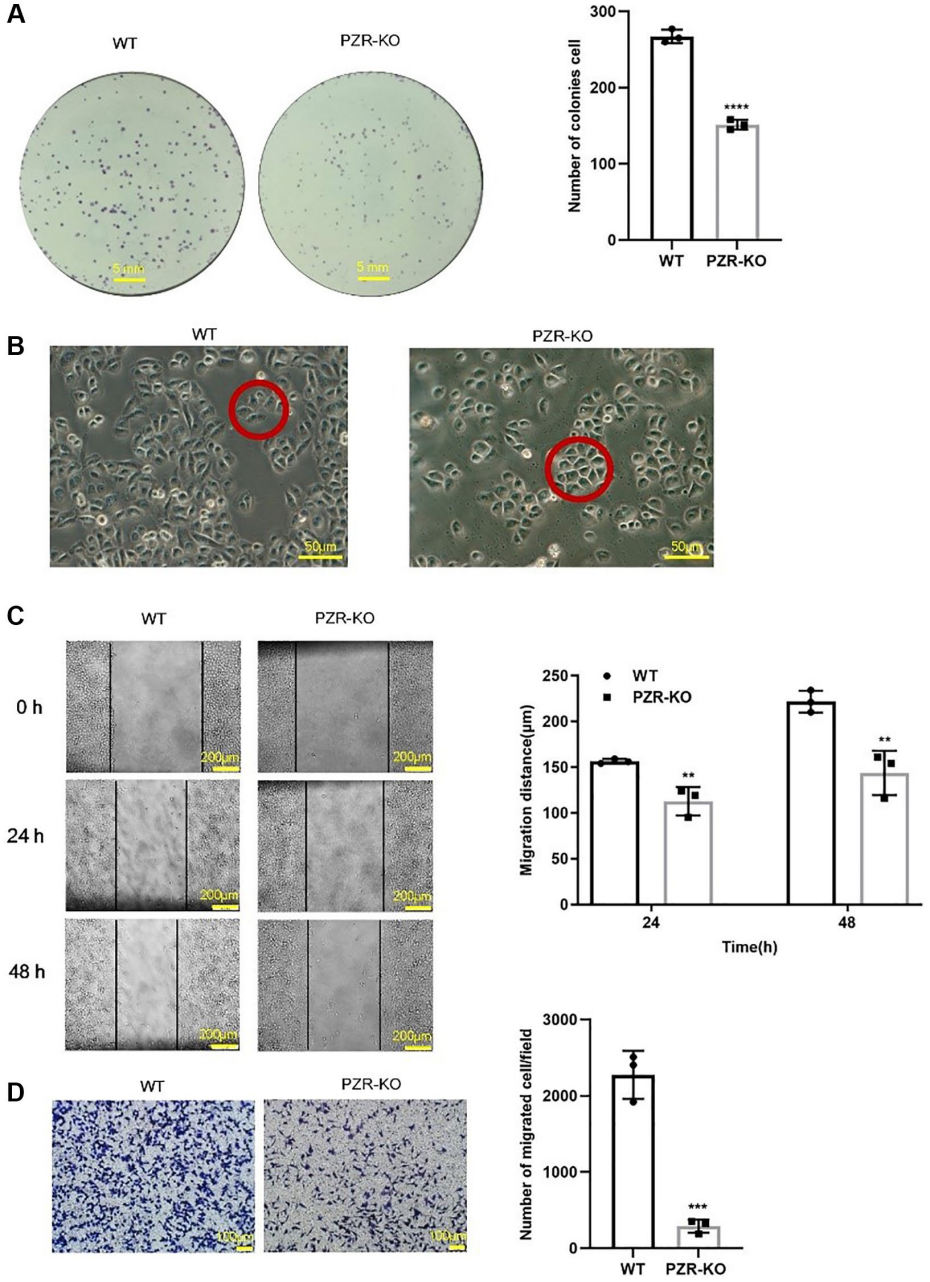
**PZR-knockout SPC-A1 cells exhibit reduced proliferative, migrating, and invading ability.** (**A**) Colonies formed by wild type and PZR-KO SPC-A1 cells were stained with crystal violet and then numerated. (**B**) Morphology of wild type and PZR-KO SPC-A1 cell (magnification, ×200). (**C**) Wound healing assays. Images show the wounded monolayers of wild type and PZR-KO SPC-A1 cells at 0 h, 24 h and 48 h (magnification, ×40). Black lines outline wound edges, and the distance between the lines was measured using the Image J software. The histogram shows migration distance after 24 h and 48 h. (**D**) Transwell invasion assays. Cells that passed through Matrigel and attached to the Transwell membrane were stained with crystal violet and numerated (magnification, ×100). All assays were done in triplicates. Data in bar graphs represent mean ± SD (*n* = 3). ^**^P < 0.01, ^***^P < 0.001.

To investigate the effects of *PZR* knockout on cell proliferation, colony formation assays were performed. The data demonstrated that knockout of PZR reduced colony-forming ability of SPC-A1 cells by 50% (Figure 3A). We also noticed a trivial change in the morphology of PZR-knockout SPC-A1 cells grown in monolayer cell culture. While the wild type cells have an elongated shape and spread out well, the PZR-knockout SPC-A1 cells are somewhat cobblestone-like with close cell-cell contacts (Figure 3B). This may be caused by reduced cell migration ability. To find out if this is the case, we employed wound healing assays. As shown in Figure 3C, knockout of PZR decreased the migration rate of SPC-A1 cells. Impaired cell migration may also affect cancer cell invasion because migration is a prerequisite for invasion. Indeed, cell invasion assays with Matrigel matrix-coated Transwell chambers demonstrated that PZR-KO cells had much lower invasion capacity than wild-type cells (Figure 3D).

### Overexpression of PZR promotes proliferation, migration, and invasion of SPC-A1 cells

**Figure 4:**
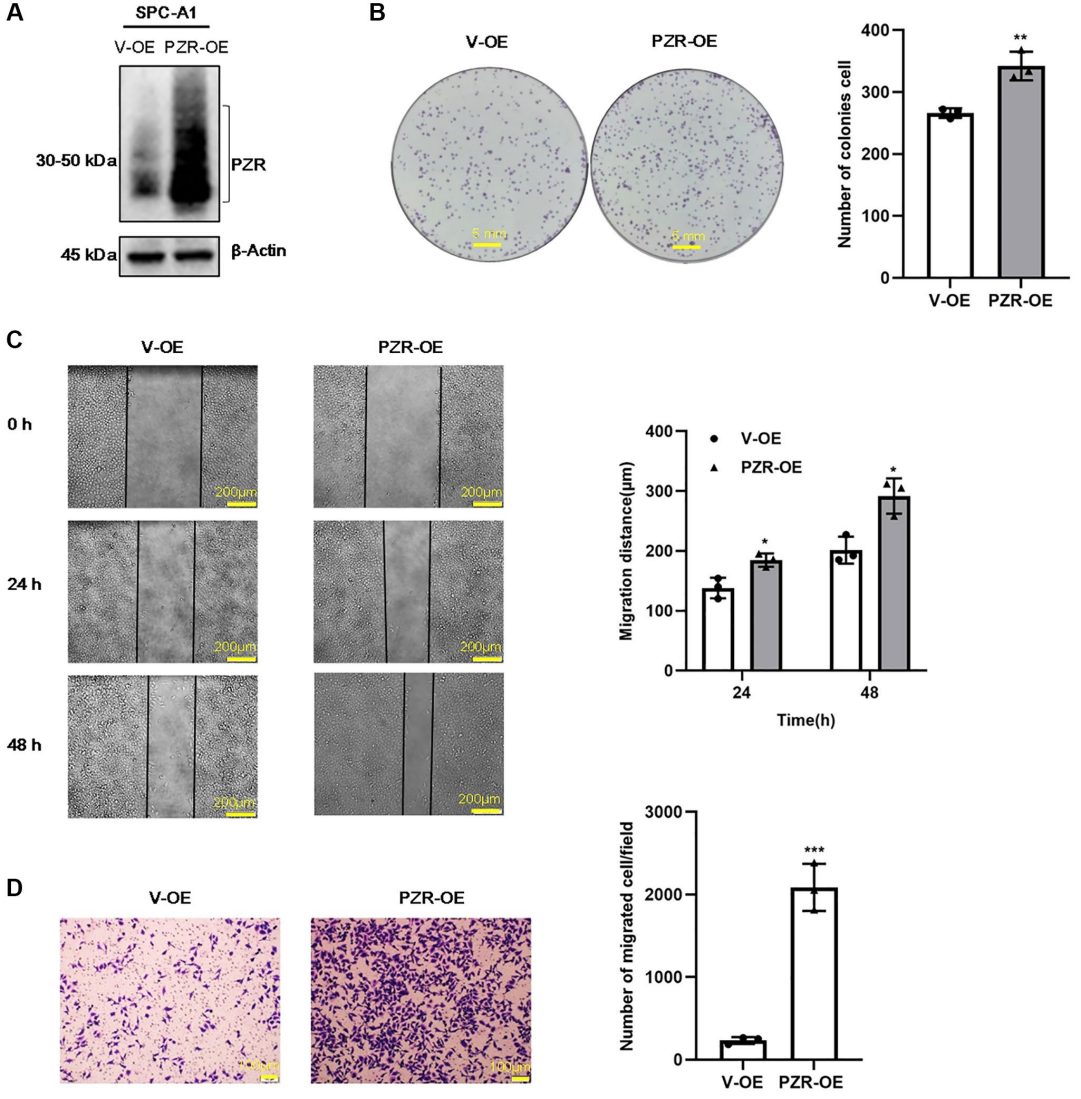
**PZR overexpression promotes proliferation, migration, and invasion of SPC-A1 cells.** SPC-A1 cells were infected with recombinant lentiviruses carrying PZR (PZR-OE) or the empty vector (V-OE), and stable cells were selected by treatment with puromycin. (**A**) Western blotting verified overexpression of PZR. (**B**) PZR-OE SPC-A1 cells showed increased colony-forming ability. (**C**) Wound healing assays demonstrated accelerated migration of PZR-OE SPC-A1 cells. Images showed the wounded monolayers of V-OE and PZR-OE SPC-A1 cells at 0 h, 24 h and 48 h (magnification, ×40). (**D**) Transwell invasion assays with Matrigel revealed enhanced penetration ability of PZR-OE SPC-A1 cells (magnification, ×100). All assays were done in triplicates. Data in bar graphs represent mean ± SD (*n* = 3). ^*^P < 0.05, ^**^P < 0.01, ^***^P < 0.001.

To verify further the effects of PZR on cellular activities, we overexpressed PZR in SPC-A1 cells by using recombinant lentivirus carrying PZR and selected PZR-overexpressing stable cell lines. Figure 4A shows marked overexpression of PZR in a typical PZR-OE cell line in comparison with the cells c control cells (V-OE). We further carried out colony-forming, migration, and invasion assays as described for the PZR-OE cells (Figure 4B–4D). In contrast to the effects observed with V-OE cells, overexpression of PZR significantly increased cell proliferation, migration, and invasion. This provides further evidence that PZR has an important role in proliferation, migration, and invasion of SPC-A1 cells.

### Knockout of PZR suppresses tumorigenicity of SPC-A1 cells *in vivo*

**Figure 5:**
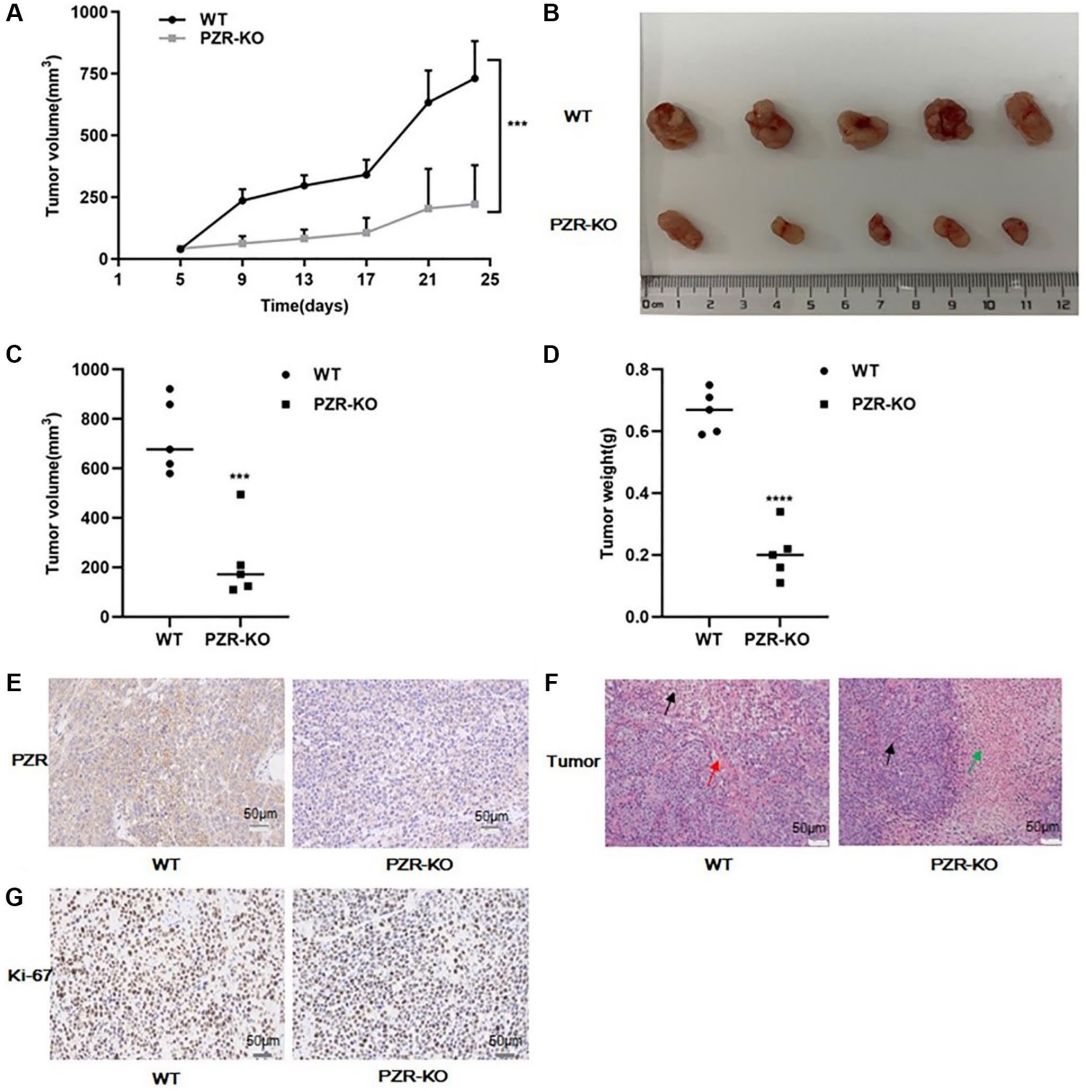
**PZR knockout reduced tumor-forming ability of SPC-A1 cells in immunodeficient NYG mice.** NYG mice (7-week male, 5 in each group) were subcutaneously engrafted with wild- type or PZR-KO SPC-A1 cells. (**A**) Tumor volumes were measured every four days from day 5. Tumor sizes are expressed as mean ± SD. (*n* = 5). ^***^P < 0.001 versus control group after day 9. (**B**–**D**) Size and weight of tumor tissues excised from NYG mice after 24 days of implantation. Error bars denote standard deviation (*n* = 5). ^***^P < 0.001, ^****^P < 0.0001. (**E**–**G**) Histochemical staining of paraffin-embedded tissue sections of tumors revealed that PZR-KO SPC-A1 cells displayed an absence of PZR expression in tumor cells (**E**), increased necrosis (green arrow) and lack of blood vessels (red arrows) (**F**), and reduced number of Ki-67-positive cells with PZR-KO SPC-A1 cells (**G**), (magnification, ×200).

We further employed immunodeficient NYG mice to test tumor formation ability of PZR-KO SPC-A1 cells *in vivo*. Generated by knocking out the expressions of Prkdc and IL2rg in NOD mice using the CRISPR genome editing technique, these mice lack T, B, and NK cells and are ideal for engraftment of human cancer cells. We subcutaneously implanted wild-type and PZR-KO SPC-A1 cells each in 5 NYG mice. Tumor development was followed for 24 days before the termination of the mice for endpoint experiments. Both wild-type and PZR-knockout SPC-A1 cells formed measurable tumors on the 5th day but the PZR-knockout cells gave rise to slower tumor growth (Figure 5A). In the end, the difference in tumor sizes was dramatic (Figure 5B). The average volume and weight of tumors from PZR-knockout SPC-A1 cells were only 221 mm^3^ and 0.2 g, respectively, while those from the wild type cells were 721 mm^3^ and 0.68 g, respectively (Figure 5C, 5D). The data clearly demonstrated that loss of PZR suppressed the growth of SPC-A1 cells *in vivo*, which agrees with the *in vitro* cell growth data described above (Figure 3A). We further performed immunohistochemical staining of tumor tissue sections. In line with expectations, PZR staining is essentially absent in tumors formed by PZR-KO SPC-A1 cells (Figure 5E). The H/E staining revealed that the PZR-KO tumors had many necrotic cells and lacked blood vessels and interstitial extracellular matrix (Figure 5F). For further assessment of tumor cell aggressiveness, we performed Ki67 staining. The data shown in Figure 5G illustrated a much lower percentage of positive cells in tumor formed by PZR-KO SPC-A1 cells.

### Altered expression of PZR in SPC-A1 cells affects tyrosine phosphorylation of c-Src, FAK, and cortactin

**Figure 6:**
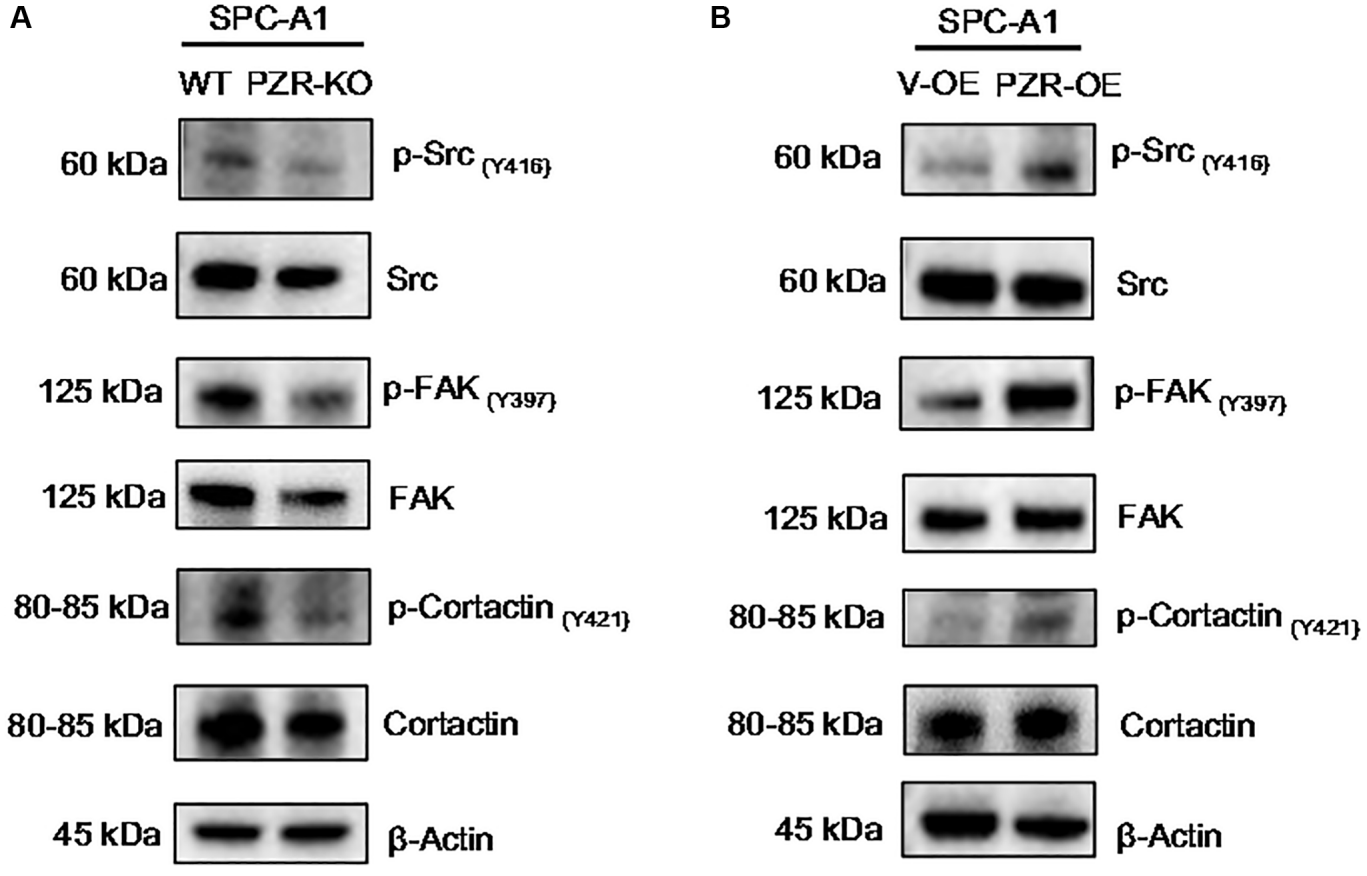
**Altered expressions of PZR affect the phosphorylation of multiple proteins involved in cell adhesion and migration.** Wild type (WT), PZR-knockout (PZR-KO), vector-overexpressing (V-OE), and PZR-overexpressing (PZR-OE) SPC-A1 cells were extracted in the RIPA buffer, and cells extract were subjected to Western blotting with the indicated antibodies. Note that knockout of PZR in SPC-A1 cells decreased phosphorylation of Src (Y416), FAK (Y397), and cortactin (Y421) (**A**) while overexpression of PZR had the opposite effects (**B**).

The data described above demonstrate that PZR plays a crucial role in cell growth, migration, and invasion *in vitro* and *in vivo*. We thought that the underlying molecular mechanism might involve cell signaling and adhesion molecules. In fact, previous studies have shown that knockdown of PZR reduced phosphorylation of focal adhesion kinase (FAK) [22], c-Src [23] and cortactin [24] that are known to be important for cell adhesion and migration. Consistent with these results, our data indicated that knockout of PZR resulted in reduced tyrosine phosphorylation of FAK, c-Src and cortactin in SPC-A1 cells (Figure 6A). Moreover, by using PZR-overexpressing SPC-A1 cells, we further demonstrated that overexpression of PZR increased tyrosine phosphorylation of FAK, c-Src and cortactin (Figure 6B). It should be noted that phosphorylation of c-Src at Y416 and FAK are indicative of their activation. FAK and c-Src form a dual kinase complex and phosphorylate various downstream proteins. One of the substrates of c-Src is cortactin, an actin filament-binding protein with an important role in cytoskeleton restructuring, cell motility, and metastasis [25–29]. The present study indicates that PZR plays an essential role in phosphorylation and activation of cortactin in SPC-A1 cells.

### Altered expression of PZR in SPC-A1 cells affects oxidative stress in SPC-A1 cells

**Figure 7:**
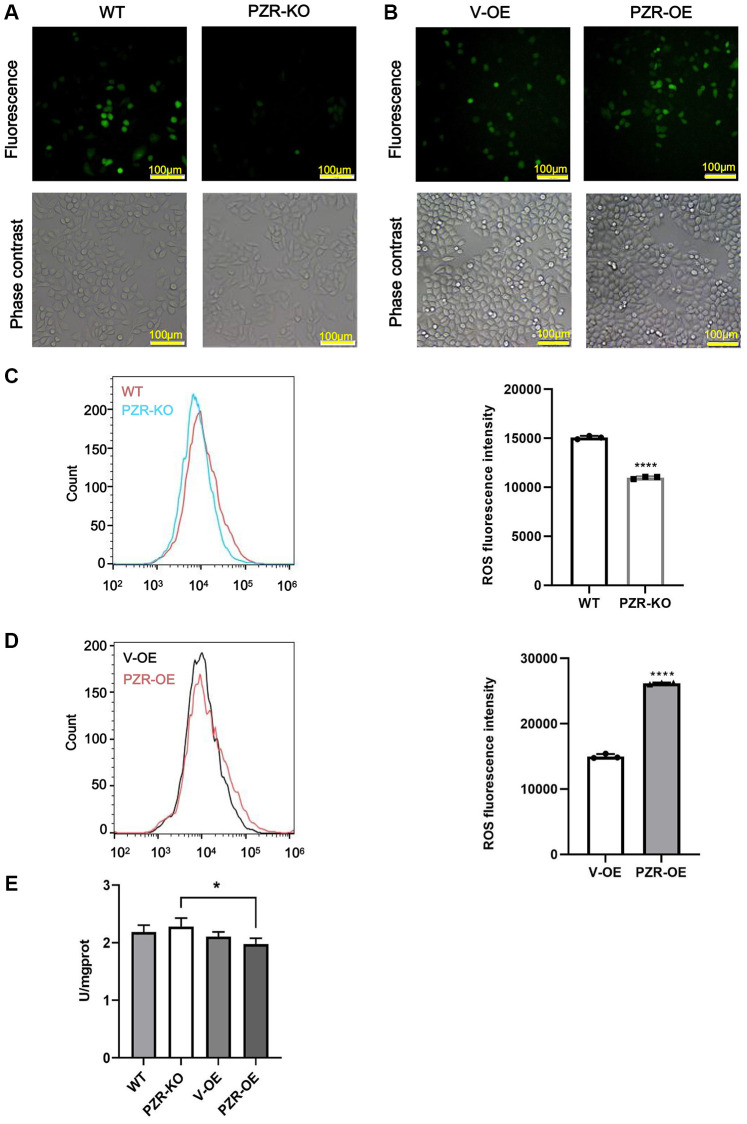
**Altered expressions of PZR affect intracellular ROS levels.** Wild type (WT), PZR-knockout (PZR-KO), vector-overexpressing (V-OE), and PZR-overexpressing (PZR-OE) SPC-A1 cells were incubated with 10 μM DCFH-DA for 30 min at 37°C in the dark and then viewed under a fluorescence microscope (**A**, **B**) or analyzed using a flow cytometer (**C**, **D**). Bar graphs show mean flow cytometric fluorescence intensity (magnification, ×100). All assays were done in triplicates. Data in bar graphs represent mean ± SD (*n* = 3). ^****^P < 0.0001. (**E**) Activities of T-SOD in SPC-A1. ^*^P < 0.05.

Previous studies have demonstrated that SHP-2 is highly susceptible to inactivation by ROS [7, 8]. By serving as a substrate and regulatory anchor protein of SHP-2, PZR may also be involved in cellular responses to oxidation stress. Notably, by performing DCFH-DA assays to measure intracellular ROS, we found that knockout of PZR significantly attenuated ROS generation in SPC-A1 while overexpression of PZR had the opposite effects as demonstrated by the results of fluorescence microscopic and flow cytometric analyses (Figure 7A–7D). The data suggest that PZR plays a role in regulating oxidation stress, which may be partly responsible for its tumor-promoting activities. Total superoxide dismutase (T-SOD) activity was assayed using the xanthine/xanthine oxidase method based on the production of O^2−^ anions. As shown in Figure 7E, the activity of T-SOD enzyme significantly decreased after overexpression of PZR compared with knockout of PZR.

### ROS inhibition by NAC reduced phosphorylation of FAK, and cortactin

**Figure 8:**
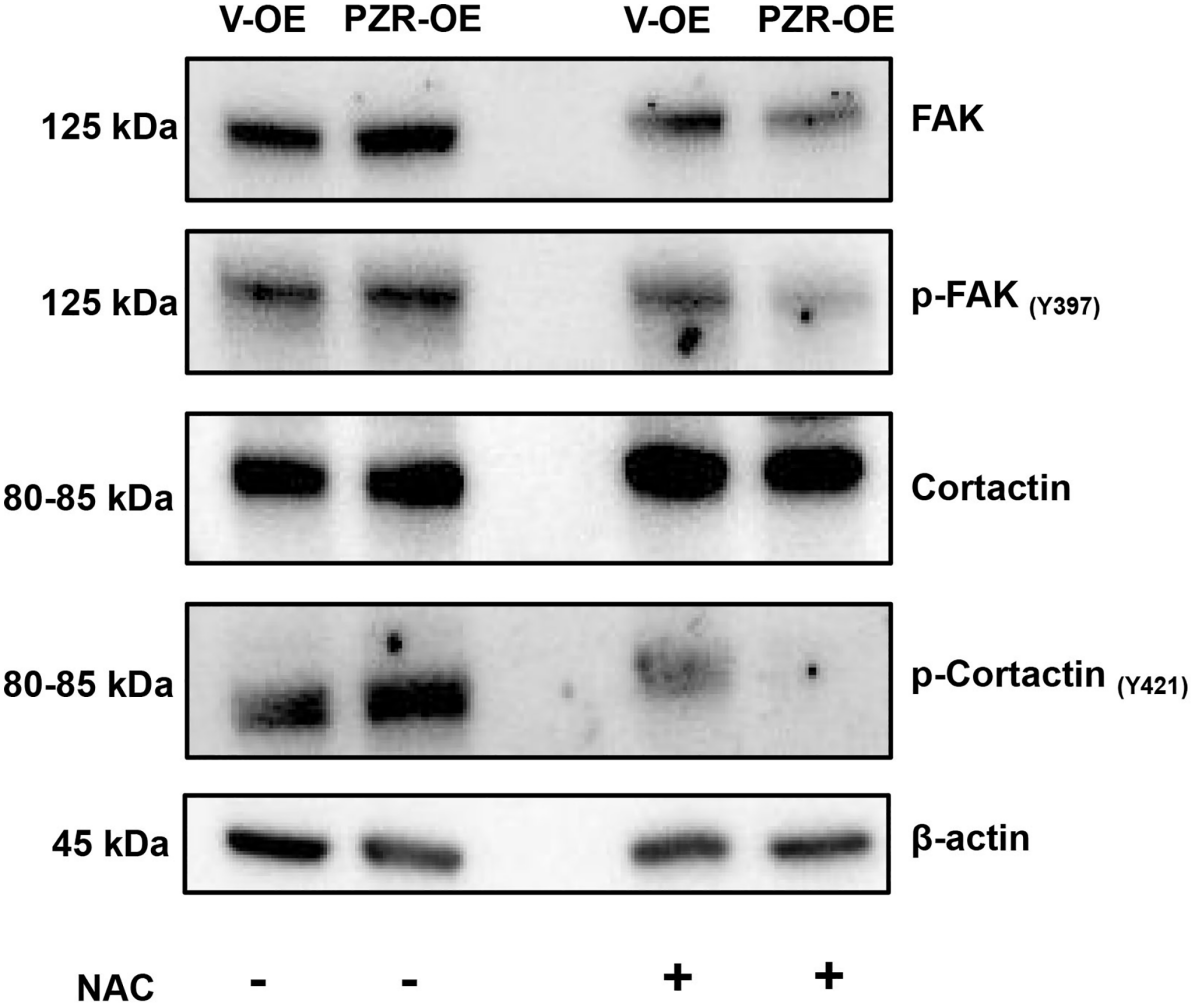
**ROS inhibition by NAC reduced phosphorylation of multiple proteins involved in cell adhesion and migration.** Vector-overexpressing (V-OE), and PZR-overexpressing (PZR-OE) SPC-A1 cells were treated with NAC (100 μM) for 1 h and then extracted in the RIPA buffer. Cells extract were subjected to Western blotting with the indicated antibodies.

N-Acetyl-Cysteine (NAC), a membrane-permeable cysteine precursor, is used as a ROS scavenger and a potent antioxidant. Cells were treated with NAC (100 μM) for 1 h. The Figure 8 shows that after treatment with NAC, tyrosine phosphorylation of FAK and cortactin decreased in PZR-OE cells.

## DISCUSSION

In the present study, we first found that high PZR expression predicts poor survival in lung cancer patients and that PZR is overexpressed in lung cancer cells. By using lung adenocarcinoma SPC-A1 cells as a model system, we further demonstrated that loss of PZR function suppressed cancer cell activities *in vitro* and *in vivo*. Our data demonstrate an important role of PZR in lung cancer development. This may have important clinical implications. First, PZR may serve as a biomarker for lung prognosis thereby guiding lung cancer treatment. Second and more importantly, PZR may serve as a target for therapeutic interventions because antibodies and soluble forms of PZR containing the extracellular region of PZR may disrupt PZR-mediated cell signaling thereby inhibiting cancer cell proliferation and metastasis. PZR are mainly modulated by SHP-2 and the SHP-2 is degraded by oxidative stress, thereby the oxidative stress status was also investigated in our research.

By generating PZR-knockout SPC-A1 cell lines using CRISPR/Cas9 technique, our data basically confirmed earlier studies on the function of PZR in cancer development by using shRNA-mediated knockdown of PZR expression. Previous studies have demonstrated that knockdown of PZR reduced proliferation and/or migration of hepatocellular carcinoma and ovarian cancer cancers [14, 19, 20]. Interestingly, a recent study found that PZR knockdown impaired the metastatic ability rather than tumor growth of A549 lung adenocarcinoma cancer cells in nude mice [30]. We observed a significant tumor growth suppression with PZR-knockout SPC-A1 but did not find any metastasis of the tumor cells into the lung with the NYG mice (data not shown). Differences in cell lines, methods of PZR expression silencing, and mouse models may explain the discrepancies.

When implanted in NYG immunodeficient mice, PZR-KO SPC-A1 cells showed reduced tumor-forming ability. Histochemical staining revealed increased necrosis accompanied by reduced blood vessels. This suggests that PZR may have a role in initiating angiogenesis by regulating interactions of various cells involved in the process. This is worthy of further investigation. Our PZR-knockout and PZR-overexpressing cell lines provide an excellent cell system for further studies in this area.

Our study indicates that PZR plays a crucial role in regulating cell migration and invasion thereby modulating cancer cell growth *in vitro* and *in vivo*. This is likely mediated by controlling the activities of FAK and c-Src. FAK and c-Src form a dual kinase complex and activate each other in adhesion-initiated cell signaling. Earlier studies demonstrated that c-Src was constitutively associated with PZR and was activated upon treatment of cells with lectin concanavalin A [2]. Furthermore, concanavalin A-induced tyrosine phosphorylation of PZR was inhibited Src inhibitors, and Src can directly phosphorylate PZR [31], thereby forming a feedback regulatory loop. It is conceivable that loss of PZR results in impaired activation of c-Src and associated signaling transduction. It will be important to define further the interplay of PZR with FAK and c-Src.

Oxidative stress, defined as over-production of ROS, has been linked to various human diseases including cancer [7]. Cancer cells exhibit aberrant redox homeostasis, and ROS are generally considered to be pro-tumorigenic as high levels of ROS are beneficial to tumor cells to maintain their proliferation while effective removal of excessive ROS by anti-oxidants inhibits tumor cell growth [32]. Our data suggest that PZR plays a role in maintaining the level of intracellular ROS in SPC-A1 cells, which is consistent with its pro-tumorigenic. This may be related to the function of SHP-2 which is regulated by ROS. It will be important to define how PZR regulates oxidative stress.
